# Association of changes of retinal vessels diameter with ocular blood flow in eyes with diabetic retinopathy

**DOI:** 10.1038/s41598-021-84067-2

**Published:** 2021-02-25

**Authors:** Yoshitaka Ueno, Takeshi Iwase, Kensuke Goto, Ryo Tomita, Eimei Ra, Kentaro Yamamoto, Hiroko Terasaki

**Affiliations:** 1 Department of Ophthalmology, Nagoya University Graduate School of Medicine, 65 Tsurumai-cho, Showa-ku, Nagoya, 466-8560 Japan; 2 Department of Ophthalmology, Akita University Graduate School of Medicine, Akita, Japan

**Keywords:** Diseases, Medical research

## Abstract

We investigated morphological changes of retinal arteries to determine their association with the blood flow and systemic variables in type 2 diabetes patients. The patients included 47 non-diabetic retinopathy eyes, 36 mild or moderate nonproliferative diabetic retinopathy (M-NPDR) eyes, 22 severe NPDR (S-NPDR) eyes, 32 PDR eyes, and 24 normal eyes as controls. The mean wall to lumen ratio (WLR) measured by adaptive optics camera was significantly higher in the PDR groups than in all of the other groups (all *P* < 0.001). However, the external diameter of the retinal vessels was not significantly different among the groups. The mean blur rate (MBR)-vessel determined by laser speckle flowgraphy was significantly lower in the PDR group than in the other groups (*P* < 0.001). The WLR was correlated with MBR-vessel (*r* = − 0.337, *P* < 0.001), duration of disease (*r* = 0.191, *P* = 0.042), stage of DM (*r* = 0.643, *P* < 0.001), systolic blood pressure (*r* = 0.166, *P* < 0.037), and presence of systemic hypertension (*r* = 0.443, *P* < 0.001). Multiple regression analysis demonstrated that MBR-vessel (β = − 0.389, *P* < 0.001), presence of systemic hypertension (β = 0.334, *P* = 0.001), and LDL (β = 0.199, *P* = 0.045) were independent factors significantly associated with the WLR. The increased retinal vessel wall thickness led to a narrowing of lumen diameter and a decrease in the blood flow in the PDR group.

## Introduction

The prevalence of diabetes mellitus has been increasing, and diabetic retinopathy (DR) has become one of the leading causes of blindness in developed countries. DR can be classified into five stages; a first stage of no apparent retinopathy, a second stage of mild non-proliferative diabetic retinopathy (M-NPDR), a third stage of moderate NPDR, a fourth stage of severe NPDR (s-NPDR), and a fifth stage of proliferative diabetic retinopathy (PDR)^1^.

DR is a metabolic disorder that is associated with hyperglycemia which causes an increase in the synthesis and accumulation of basement membrane components. These changes result in a thickening of the basement membranes of blood vessels^2^. Several studies of fundus photographs showed microvascular changes in the blood vessels of patients with diabetes^3–7^. The diameter of the retinal vessels is important because it may predict the progression of the DR, and the results of two studies reported that there were beneficial effects of photocoagulation for macula edema and proliferative retinopathy^3, 4^. The severity of the retinopathy and renal dysfunction were found to be associated with the degree of retinal arteriolar narrowing in patients with type1 diabetes^5^. This narrowing is important because it is related to the incidence of strokes and cardiovascular disease in the general population and in individuals with diabetes^6, 7^. The authors of these studies concluded that there was a significant association between the retinal vessel diameter and systemic complications. However, these studies could not assess the changes in the thickness of the retinal vessel wall because of the low resolution of conventional fundus cameras used to record the images of the retinal blood vessels.

The wall to lumen ratio (WLR) is the ratio of the vascular wall thickness to the luminal diameter, and this ratio can be used as an in vivo parameter of arterial remodeling. This parameter is important in the field of vascular medicine because it suggests the degree of vascular stenosis and is predictive of end-organ damage^8–10^. The WLR can increase due to wall thickening, or by a narrowing of the lumen, or a combination of both. Thus, arterial remodeling is best characterized by an increase in the WLR.

Adaptive optics (AO) technology can allow a noninvasive recording of high resolution images of the photoreceptors and retinal vessel walls by correcting the optical wavefront aberrations^11^. Recently, studies have evaluated the retinal vessels including wall thickness and the WLR in detail using the images obtained by an AO camera^12–17^.

Measurements of the ocular blood flow in diabetic retinopathy are necessary to understand the role played by alterations of the retinal hemodynamics in the progression of retinopathy and to assist in the management of the progression. Many studies have investigated the hemodynamics of patients with diabetes mellitus by various technologies including laser doppler velocimetry (LDV) and video fluorescein angiography^18–22^. However, few studies have evaluated the ocular blood flow at each stage of diabetic retinopathy. Due to the time-consuming measurements, earlier ocular blood flow measuring devices were not suitable for large scale clinical trials. Laser speckle flowgraphy (LSFG) (Softcare Co., Ltd., Fukutsu, Japan) is a method of measuring the relative blood flow in the choroid and optic nerve head (ONH) quantitatively. It requires only 4 s to obtain one image^23–25^. The LSFG values have been shown to be highly correlated with the actual blood flow values as determined by hydrogen gas clearance and the microsphere method^26, 27^. This means that the values of the variables determined with LSFG of different individuals can be compared reliably. Therefore, LSFG is believed to be suitable for evaluating retinal blood flow in a large number of patients non-invasively and rapidly.

Nevertheless, to the best of our knowledge, there has not been a study that assessed both the morphological changes of the retinal vessels and microcirculation at each stage of DR.

Thus, the purpose of this study was to evaluate the arterial wall thickness, external diameter, and lumen diameter of the retinal vessels at different stages of DR. To accomplish this, we measured the morphological parameters in the high-resolution images obtained by an AO-camera and the ocular blood flow parameters by LSFG from patients at different stages of DR.

## Patients and methods

### Ethics statement

This was a retrospective, cross-sectional, single-center study, and the procedures were approved by Institutional Review Board and the Ethics Committee of the Nagoya University Hospital (Nagoya, Japan). The study was performed at the Nagoya University Hospital, and the procedures conformed to the tenets of the Declaration of Helsinki. All subjects signed a written informed consent form. Permission was also obtained to use the data collected for future research.

### Subjects

We reviewed the medical records of all Japanese patients who were diagnosed with type 2 diabetes between April 2016 and March 2018. The normal control eyes were the fellow eyes of age-matched patients who visited to our hospital to treat the other eye for rhegmatogenous retinal detachment (RRD) or epiretinal membrane (ERM) without diabetes. None of these patients had any retinal diseases including RRD and ERM. The stage of the DR was determined by indirect ophthalmoscopy and fluorescein angiograms of the dilated eyes by two retinal specialists (YU, TI). The eyes with diabetes were divided into 4 stages; no apparent diabetic retinopathy (NDR), mild and moderate nonproliferative diabetic retinopathy (M-NPDR), severe NPDR (S-NPDR), and proliferative diabetic retinopathy (PDR)^28^. In eyes with PDR, photocoagulation was performed with a total of 1200–3500 spots and 200-µm spot sizes with pulse duration of 0.2 s to obtain a complete pan retinal photocoagulation (PRP). When both eyes met the inclusion criteria, the data of one eye were randomly selected for the statistical analyses.

### Exclusion criteria

The exclusion criterion included the presence of any macular abnormalities such as asymptomatic pigment epithelial detachment or choroidal neovascularization, use of topical anti-glaucoma medications, history of other ophthalmic disorders, use of systemic hormonal medications, or prior steroid or anti-vascular endothelial growth factor (VEGF) therapy for the diabetic macular edema at least 1 year before the measurements, and axial length (AL) > 26.5 mm^29^.

### Measurements of clinical parameters

The ALs were measured by partial optical coherence interferometry (IOLMaster; Carl Zeiss Meditec, La Jolla, CA), and the intraocular pressure (IOP) was measured with a handheld tonometer (Icare; Tiolat Oy, Helsinki, Finland). The systolic blood pressure (SBP) and diastolic blood pressure (DBP) of the left brachial artery at the height of the heart in a sitting position were measured for with an automatic sphygmomanometer (CH-483C; Citizen, Tokyo, Japan). The mean arterial blood pressure (MAP) and mean ocular perfusion pressure (MOPP) were calculated as follows: MAP = DBP + 1/3(SBP − DBP) and MOPP = 2/3MAP − IOP, respectively^30^.

The laboratory profile of each patient consisted of the hemoglobin A1c (HbA1c) level, total cholesterol (mg/dL), triglycerides (mg/dL), low-density lipoprotein-cholesterol (LDL-C mg/dL), and high-density lipoprotein-cholesterol (HDL-C mg/dL) obtained from blood samples. The HbA1c level was expressed based on the scale of the National Glycohemoglobin Standardization Program. Hypertension was defined as a SBP ≥ 140 mmHg or a DBP ≥ 90 mmHg or the use of any antihypertensive medication^31^. Dyslipidemia was defined to be present when the serum of the individual had low-density lipoprotein (LDL) cholesterol levels ≥ 140 mg/dL and/or high-density lipoprotein (HDL) cholesterol levels < 40 mg/dL and/or triglyceride values ≥ 150 mg/dL in subjects with a history of cholesterol-lowering therapy^32^.

### Laser speckle flowgraphy measurements

**Figure 1:**
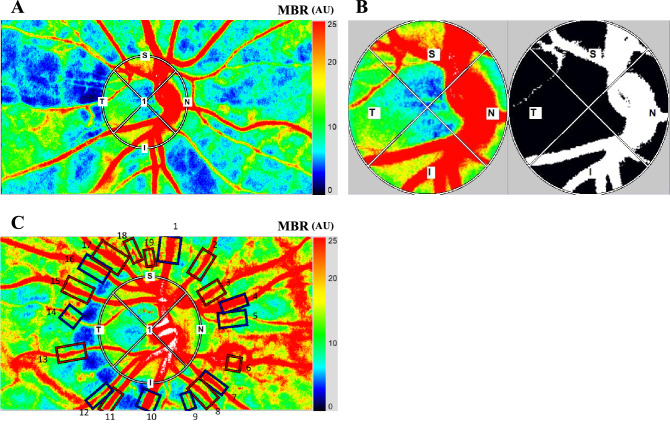
A circular marker was set surrounding the optic nerve head (ONH) to measure the mean blur rate (MBR) (**A**). The “vessel extraction’’ function of the software then identified the vessel and tissue areas on the ONH so that the MBR could be assessed separately as the vessel areas (MBR-vessel) and the tissue areas (MBR-tissue) (**B**). The rectangular areas (1 to 19) were set semi-automatically to the vessels around the optic nerve head concentrically and the total retinal flow index (TRFI) was calculated (**C**).

LSFG-NAVI (Softcare, Fukuoka, Japan) was used to determine the relative blood flow on the ONH and the retinal microcirculation. The principles of LSFG have been described in detail^33–35^. To evaluate the circulation on the ONH, a circular marker was set surrounding the ONH (Fig. 1A). The “vessel extraction’’ function of the software then identified the vessel and tissue areas on the ONH so that the MBR could be assessed separately as the vessel areas (MBR-vessel) and the tissue areas (MBR-tissue) (Fig. 1B). To evaluate the retinal circulation, the relative flow volume (RFV) index and the total retinal flow index (TRFI) were calculated. The calculation of the RFV has been described in detail^36^. The TRFI represents the total of retinal arterial and venous blood flow volume which was calculated from the total RFV index for all of the major retinal vessels around the ONH semiautomatically. To measure the TRFI, rectangular bands were set around all of the major vessels of the ONH using the built-in image analysis software (Fig. 1C). If there was an error in the placement, we corrected the location of the rectangular band manually.

LSFG was performed twice for each time point in all of the eyes. The average MBR values were calculated for each circle or rectangle using the LSFG Analyzer software (v.3.1.59).

### Adaptive optics (AO) imaging

The rtx1 AO is an adaptive optics device (Imagine Eyes, Orsay, France)^37^, and its principles have been described in detail^38, 39^. We recorded images of the arterioles in Zone B which was located 0.5–1 disc diameter from the optic disc margin. The vascular measurements of this area were made by the software AO which can detect the retinal arteries. We obtained the external diameter (ED), lumen diameter (LD), wall thickness (WT), and wall to lumen ratio (WLR) for the retinal arteries, which were measured by two retinal specialists (YU, TI).

### Statistical analyses

The value of each parameter is presented by the means ± standard deviations. Comparisons between groups were made using one-way ANOVA (for continuous variables) and the x^2^ test (for categorical variables). One-way ANOVA was followed by a post hoc comparison with Tukey HSD or Games-Howell procedure. Spearman’s rank correlation coefficient tests were used to determine the significance of the correlation coefficients between the variables. Multiple stepwise regression analysis was used to determine the association between blood flow parameters and the other variables. All statistical analyses were performed using IBM SPSS Statistics for Windows, v.24 (IBM Corp., Armonk, NY). The significance level was set at a probability (*P*) value < 0.05.

## Results

### Demographics of subjects

This study included 47 NDR eyes, 36M-NPDR eyes, 22 S-NPDR eyes, 32 PDR eyes, and 24 control eyes. The baseline clinical demographic data on all subjects are presented in Table 1. The mean duration after PRP was 4.2 ± 2.9 years in the PDR group. No significant differences were observed in the sex distribution, AL, SBP, DBP, IOP, OPP, total cholesterol, LDL, HDL, and triglyceride among the five groups. However, there were significant differences in the age (*P* = 0.012), HbA1c (*P* = 0.003), duration of diabetes (*P* = 0.003), hypertension ratio (*P* = 0.001), and dyslipidemia ratio (*P* = 0.047) among the groups.

**Table 1:** Clinical characteristics of subjects.

Characteristics	Control	NDR	M-NPDR	S-NPDR	PDR	*p*-value
N	24	47	36	22	32	–
Age (years)	67.2 ± 10.1	62.7 ± 12.9	63.4 ± 12.7	55.1 ± 12.6	59.7 ± 11.8	0.012
Sex (male:female)	11 : 13	27 : 20	21 : 15	13 : 9	16 : 16	0.797
Axial length (mm)	23.9 ± 1.1	24.0 ± 1.9	24.2 ± 1.1	24.2 ± 1.1	24.0 ± 1.0	0.832
HbA1c (%)	–	7.0 ± 0.9	7.8 ± 1.3	8.2 ± 2.0	7.3 ± 1.0	0.003
Duration of diabetes (years)	–	10.1 ± 6.9	17.6 ± 11.1	13.1 ± 7.8	17.3 ± 10.1	0.003
Systolic blood pressure (mmHg)	134.6 ± 16.5	126.6 ± 17.5	135.5 ± 25.2	131.8 ± 16.3	141.8 ± 29.7	0.079
Diastolic blood pressure (mmHg)	77.0 ± 9.4	76.1 ± 11.9	77.9 ± 12.9	78.8 ± 10.1	78.0 ± 15.9	0.902
Intra ocular pressure (mmHg)	13.0 ± 2.5	14.5 ± 2.5	14.8 ± 3.3	14.1 ± 3.4	15.2 ± 4.3	0.126
Ocular perfusion pressure (mmHg)	51.3 ± 7.2	47.5 ± 8.8	49.9 ± 10.0	50.2 ± 8.7	50.8 ± 11.2	0.436
Total cholesterol (mg/dl)	202.0 ± 35.6	178.4 ± 37.7	171.5 ± 32.9	186.2 ± 63.6	194.1 ± 37.7	0.102
Low-density lipoprotein (mg/dl)	–	102.2 ± 27.9	93.4 ± 26.1	95.0 ± 23.8	110.6 ± 32.4	0.210
High-density lipoprotein (mg/dl)	–	58.8 ± 21.7	51.6 ± 15.9	45.7 ± 13.0	50.1 ± 14.3	0.087
Triglyceride (mg/dl)	134.8 ± 92.4	128.6 ± 69.7	156.5 ± 114.7	149.7 ± 97.4	157.6 ± 82.2	0.655
Hypertension (n/%)	4/16.7	15/31.9	18/50.0	12/54.5	23/71.9	0.001
Dyslipidemia (n/%)	6/25.0	22/46.8	19/52.8	9/40.9	19/59.3	0.047

### Differences in retinal arterial parameters among groups

**Figure 2:**
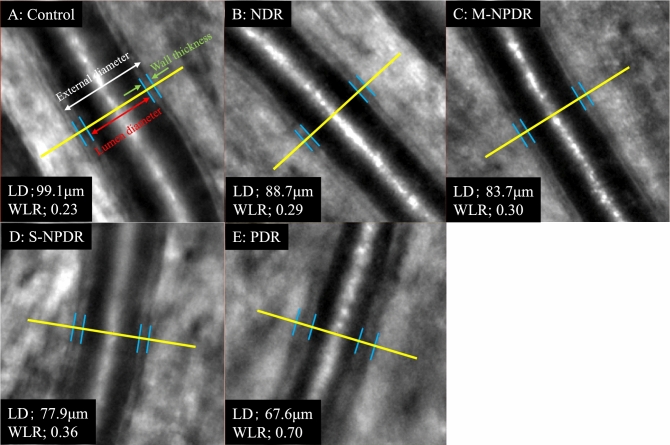
Representative images obtained with an Adaptive Optics device (images in each stages of diabetic retinopathy (DR). The images show the external diameter, the lumen diameter (LD), wall thickness, and wall to lumen ratio (WLR). Eyes in the control group (**A**); no diabetic retinopathy (NDR) group (**B**); mild or moderate non-proliferative diabetic retinopathy (M-NPDR) group (**C**); severe NPDR (S-NPDR) group (**D**); and PDR group (**E**) are shown. Eyes in the DR groups have higher WLR, especially in the PDR group.

**Figure 3:**
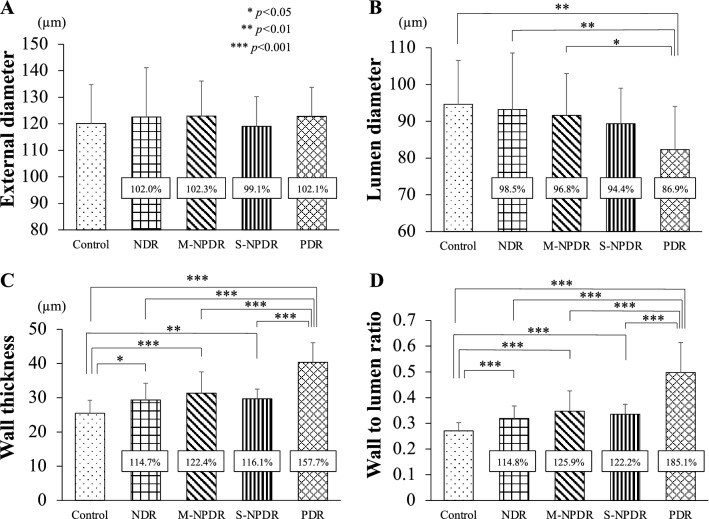
Differences in the changes in the diameter of the retinal vessels determined by AO images among the groups. The percentage change is expressed for the four groups with diabetes, and the values are compared to that of the control group. There were no significant differences in the external diameter among the groups (**A**). The lumen diameter in the PDR group is significantly smaller than in the control, the NDR, and the M-NPDR groups (**B**). The mean wall thickness (**C**) and the mean wall to lumen ratio (**D**) in the control group are significantly lower than in the other groups. The mean wall thickness (**C**) and the mean wall to lumen ratio (**D**) in the PDR groups are significantly larger than in the other groups.

The repeatability of the measurements between the graders was excellent with an ICC of 0.99 for the external diameter, 0.99 for the lumen diameter, and 0.99 for the wall thickness. There was no significant difference in the external diameter of the retinal vessels among the five groups, but the lumen diameter in the PDR group was significantly smaller than that in the control, the NDR, and the M-NPDR groups (*P* = 0.004, *P* = 0.002, *P* = 0.024, respectively; Table 2, Figs. 2, 3). The vessel wall was significantly thinner in the control group than that in the NDR, the M-NPDR, the S-NPDR, and the PDR group (*P* = 0.040, *P* < 0.001, *P* = 0.001, *P* < 0.001, respectively). In addition, the wall was significantly thicker in the PDR group than in the control, the NDR, the M-NPDR, and the S-NPDR groups (all *P* < 0.001). The mean WLR was significantly lower in the control group than in the NDR, the M-NPDR, the S-NPDR, and the PDR group (all *P* < 0.001), and it was significantly greater in the PDR group than in the NDR, the M-NPDR, and the S-NPDR groups (all *P* < 0.001).

**Table 2:** Retinal artery variables measured by AO camera.

Variables	Control	NDR	M-NPDR	S-NPDR	PDR	*p* value
External diameter (μm)	120.12 ± 14.67	122.57 ± 18.60	122.93 ± 13.20	119.06 ± 11.16	122.69 ± 11.07	0.714
Lumen diameter (μm)	94.62 ± 11.95	93.21 ± 15.37	91.60 ± 11.37	89.35 ± 9.64	82.31 ± 11.74	0.001
Wall thickness (μm)	25.58 ± 3.80	29.36 ± 4.88	31.33 ± 6.24	29.70 ± 2.82	40.35 ± 5.74	< 0.001
Wall to lumen ratio	0.27 ± 0.03	0.31 ± 0.04	0.34 ± 0.07	0.33 ± 0.03	0.50 ± 0.11	< 0.001

### Comparison of ocular blood flow among groups

**Figure 4:**
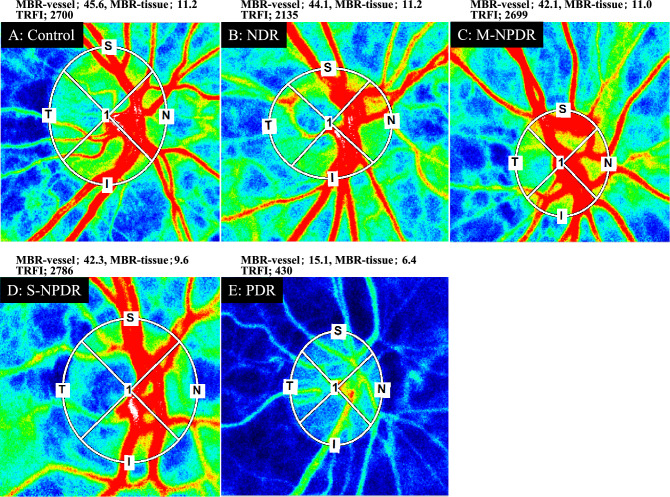
Representative composite color maps on the optic nerve head determined by LSFG at each stages of DR. Red color indicates a high mean blur rate (MBR), and blue color indicates a low MBR. Eyes in the control group (**A**); NDR group (**B**); M-NPDR group (**C**); S-NPDR group (**D**); and PDR group (**E**) are shown.

**Figure 5:**
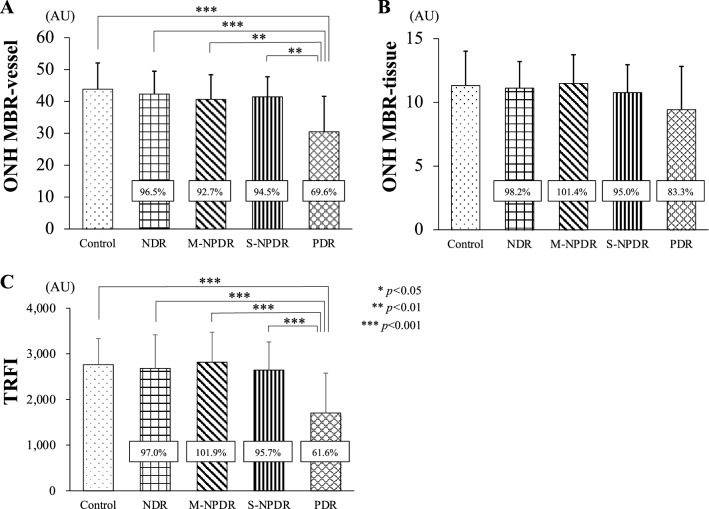
Differences in blood flow parameters determined by LSFG among the groups. The percentage differences are shown for four groups with diabetes and compared to the control group. The MBR of the vessels on the ONH in the PDR group is significantly lower than that in the other groups (**A**). There is no significant difference in the MBR-tissue among the groups (**B**). The total retinal flow index (TRFI) in the PDR group is significantly lower than the other groups (**C**).

The MBR-vessel was significantly lower in the PDR group than that in the control, the NDR, the M-NPDR, and the S-NPDR groups (all *P* < 0.001; Table 3, Figs. 4, 5). There was no significant difference in the MBR-tissue among the groups. The TRFI was significantly lower in the PDR group than that in the control, the NDR, the M-NPDR, and the S-NPDR groups (all *P* < 0.001).

**Table 3:** Parameters determined by LSFG.

Parameters	Control	NDR	M-NPDR	S-NPDR	PDR	*p* value
MBR-vessel (AU)	43.83 ± 8.22	42.30 ± 7.17	40.64 ± 7.74	41.44 ± 6.30	30.50 ± 11.09	< 0.001
MBR-tissue (AU)	11.31 ± 2.69	11.11 ± 2.08	11.47 ± 2.25	10.75 ± 2.19	9.42 ± 3.39	0.099
TRFI (AU)	2764 ± 572.2	2682 ± 736.1	2817 ± 657.6	2644 ± 617.0	1704 ± 873.5	< 0.001

AU: arbitrary units, TRFI: total retinal flow index.

### Correlation of vascular variables to other parameters

**Figure 6:**
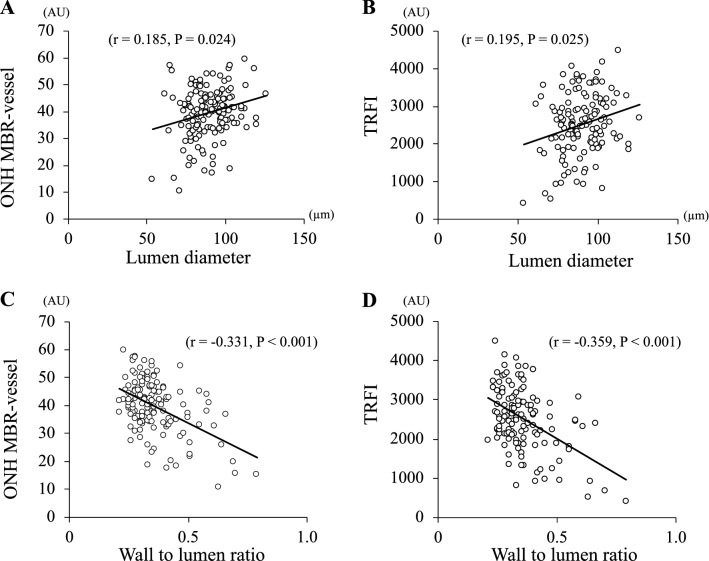
Correlation of morphological variables with blood flow parameters. The lumen diameter is significantly correlated with the MBR-vessel (**A**) and TRFI (**B**). The WLR is correlated with the MBR-vessel (**C**) and TRFI (**D**).

The lumen diameter was significantly correlated with the MBR-vessel (*r* = 0.185, *P* = 0.024), the TRFI (*r* = 0.195, *P* = 0.025), and the stage of DR (*r* = − 0.288, *P* < 0.001; Table 4, Fig. 6). The wall thickness was significantly correlated with the MBR-vessel (*r* = − 0.331, *P* < 0.001), -tissue (*r* = − 0.172, *P* = 0.036), the TRFI (*r* = − 0.362, *P* < 0.001), the duration of disease (*r* = 0.223, *P* = 0.017), the stage of DR (*r* = 0.587; *P* < 0.001), SBP (*r* = 0.195, *P* = 0.014), and the presence of systemic hypertension (*r* = 0.400, *P* < 0.001). The WLR was significantly correlated with the MBR-vessel (*r* = − 0.337, *P* < 0.001), the TRFI (*r* = − 0.359, *P* < 0.001), the duration of disease (*r* = 0.191, *P* = 0.042), the stage of the DR (*r* = 0.643, *P* < 0.001), the SBP(*r* = 0.166, *P* < 0.037), and the presence of systemic hypertension(*r* = 0.443, *P* < 0.001).

**Table 4:** Spearman’s rank correlation coefficient of vascular variables with other parameters.

Variables	MBR-vessel	MBR-tissue	TRFI	Age	Duration of disease	DR stage	HbA1c	T-chol	LDL	HDL	TG	SBP	DBP	OPP	HT	DL
External diameter (μm)	0.012	− 0.012	0.036	0.121	0.437	0.074	0.020*	− 0.136	− 0.007	− 0.205	− 0.107	0.038	− 0.058	− 0.020	0.043	0.100
Lumen diameter (μm)	0.185*	0.085	0.195*	0.110*	− 0.012	− 0.288***	0.117	− 0.128	− 0.020	− 0.140	− 0.156	− 0.033	− 0.092	− 0.038	− 0.149	0.009
Wall thickness (μm)	− 0.331***	− 0.172*	− 0.362***	0.042	0.223*	0.587***	0.024	− 0.100	0.062	− 0.181	− 0.002	0.195*	0.081	0.074	0.400***	0.116
Wall to lumen ratio	− 0.337***	− 0.150	− 0.359***	− 0.023	0.191*	0.643***	− 0.066	− 0.032	0.022	− 0.096	0.097	0.166*	0.105	0.069	0.443***	0.085

ED: external diameter; LD: lumen diameter; WT: wall thickness; WLR: wall to lumen ratio; T-chol: total cholesterol; LDL: low density lipoprotein; HDL: high density lipoprotein; TG: triglyceride; SBP: systolic blood pressure; DBP: diastolic blood pressure; OPP: ocular perfusion pressure; HT: hypertension; DL: dyslipidemia.

*p < 0.05, **p < 0.01, ***p < 0.001.

Stepwise multiple regression analysis showed that the WLR was an independent factor that was significantly associated with the MBR-vessel (β = − 0.389, *P* < 0.001), the presence of hypertension (β = 0.334, *P* = 0.001), and LDL (β = 0.199, *P* = 0.045) (Table 5). In addition, after excluding the PDR group from the all data, multiple regression analysis also showed the WLR was an independent factor that was significantly associated with the presence of hypertension (β = 0.273, *P* = 0.001) and the MBR-vessel (β = − 0.207, *P* = 0.014).

**Table 5:** Results of multiple regression analysis for independence factors contributing to wall to lumen ratio.

Dependent	Independent	*β*	*p*-value
Wall to lumen ratio	MBR-vessel	− 0.389	< 0.001
	Hypertension	0.334	0.001
	Low-density lipoprotein	0.199	0.045
	Systolic blood pressure	0.141	0.156
	Age	− 0.121	0.217
	Diastolic blood pressure	0.115	0.238
	Dyslipidemia	− 0.072	0.487
	HbA1c	0.045	0.648
	Triglyceride	0.041	0.688
	Duration of disease	0.033	0.747
	Total-cholesterol	0.015	0.902
	High-density lipoprotein	0.005	0.958

## Discussion

Our results showed that the high-resolution images obtained by the AO-camera allowed us to measure the different morphological parameters of the retinal arteries. In addition, LSFG allowed us to measure the relative blood flow. These measurements were made in patients at different stages of DR, and the results allowed us to determine whether significant correlations were present between the morphological and blood flow characteristics of the retinal arterial vessels and the stage of the DR.

Our results showed that the wall thickness and the WLR were significantly different among the five groups, and it was greater even in the NDR group than in the control group. The results of a large clinical trial showed that patients with type 2 diabetes without retinopathy had smaller mean arteriolar and venular lumen diameters as determined by fundus camera than non-diabetic individuals^40^. However, the external diameter of the vessels could not be measured accurately because of the low resolution conventional fundus cameras. Arichika et al. examined the retina of patients with DR by AO scanning laser ophthalmoscopy (SLO) and reported that the retinal arterial walls were significantly thicker in diabetic patients without clinically apparent DR than in the control^12^. However, the lumen and the external diameter was not significantly different. In addition, Zaleska-Zmijewska et al. reported that the vessel wall thickness and the WLR in the NDR group was thicker than that of the control group using the images obtained by an AO-camera, but the lumen and the external diameter were not significantly different^16^. Our results confirm these findings.

It has been suggested that the sclerosis of the vascular wall in diabetic patients was due in part to stiffening of the collagen caused by advanced glycosylated products with collagen cross-linking and elastin degeneration^41, 42^. Also, the lumen of the retinal arteries have been reported to become narrower due to the growth of smooth muscle cells and vascular fibrosis^43^. This would then contribute to an increase in the WLR in eyes of diabetic patients^44, 45^. Interestingly, there was no significant difference in the external diameter among the five groups. These results imply that these morphological changes in eyes of diabetic patients result from a thickening of the vessel wall and a narrowing of the lumen diameter while not increasing the external diameter even in eyes treated by laser photocoagulation.

A thickening of the wall, a narrowing of the lumen, or a combination of both can cause an increase in the WLR. In addition, the images of arterioles were taken 0.5 to 1 disc diameter from the optic disc margin. The measured values were not very different, i.e., small standard deviations, but varied depending on the vessel. Accordingly, the WLR should be a more accurate variable to compare the morphological differences among the groups.

On the other hand, there were no differences in the blood flow (MBR) and blood flow velocity (TRFI) determined by LSFG among the NDR, M-NPDR, and S-NPDR groups and the control group. In a prospective study of patients with type 1 disease showed that the blood flow determined by laser doppler velocimetry (LDV) was initially decreased in the retinal arterioles and was then increased with long follow-up periods^46^. Another study reported that the retinal blood flow was lower in patients with type 2 diabetes and early DR^19^. Recently, Palochak et al. reported that the blood flow in eyes with DM without DR determined by AO-SLO was significantly higher than controls which contrasted with the significantly decrease in blood flow in eyes with NPDR^47^.

There are several explanations for the finding that the blood flow results are not consistent in eyes with untreated diabetic retinopathy^19, 46, 47^. First, it has been reported that the retinal blood flow is increased while vessel density is decreased as determined by OCTA in eyes with NDR^48^. These findings suggest that the increased blood flow is a temporary compensatory mechanism for the reduced blood vessel density to meet the metabolic demand of the photoreceptors. Second, it is known that the photoreceptors are the most oxygen consuming tissue in the retina and the number of photoreceptor is reduced in NPDR^16^. The characteristics would suggest that the more advanced retinopathy led to a greater reduction of blood flow. Because the sample size was relatively small in this study and a significant difference was not detected, it might be that the blood flow is reduced depending on the grade of retinopathy in the case where a larger number of subjects were examined. Third, the features of the measurement device would cause the difference in the results and LSFG cannot detect the difference in blood flow parameters among the control and untreated diabetic retinopathy group. Shiba et al. reported that the MBR of the ONH in diabetic patients without retinopathy did not differ from that of nondiabetic patients^49^. Fourth, the level of VEGF expression might influence the results of the blood flow measurements. The injection of anti-VEGF agents has been reported to reduce the ocular blood flow in patients with diabetic retinopathy^50^ indicating that retinal blood flow is affected by VEGF. The concentration of VEGF would be not consistent even in the same grade of retinopathy, which might be related to our results. This question can only be answered by longitudinal, large scale, multimodal imaging studies.

Our results showed significant thicker walls resulting in narrower lumen diameters and higher WLR in the PDR group compared to the other four groups. These results indicate that the presence of another mechanism because all of the eyes in the PDR group underwent photocoagulation treatments. Several studies have reported that photocoagulation leads to a narrowing of the retinal arteries and veins using conventional fundus photography^5, 40, 51–53^. In addition, the MBR-vessel and the TRFI were significantly reduced in the PDR group compared to the other groups. These results are in good agreement with the morphological changes, and photocoagulation destroys the ischemic tissue including the photoreceptors, reduces the oxygen demand, and improves the oxygenation of the inner retina. These changes would lead to an autoregulatory vasoconstriction and reduced retinal blood flow^54^. An animal study showed an increase in the oxygen delivered from the choroidal circulation to the inner retina after photocoagulation^55^. Actually, earlier studies reported a decrease in ocular blood flow after PRP^52, 56–59^. Furthermore, the negative correlation between the ocular blood flow and the WLR in our study suggests that the vasoconstriction of retinal artery after photocoagulation is related to the increase of vessel wall thickness and narrowed lumen diameter secondary to the decrease of ocular blood flow although external diameter did not change so much.

Multiple regression analysis showed that the WLR were significantly correlated with the MBR of the vessels even when the PDR group was excluded. There have been reports about the relationship between blood flow and vessel morphological changes. Physiologically, this might be explained by wall shear stress (WSS) playing a role in maintaining the relation between arterial diameter and blood velocity^60–64^. And metabolically, this might be due to factors such as endothelin-1. Khuu et al. reported elevated level of endothelin-1 in aqueous humor and reduced blood flow in patients with mild-to-moderate NPDR^65^. These factors might be the physiological and metabolic parameters explaining the correlation between retinal blood flow index and lumen diameter.

Our results showed that a history of hypertension was correlated with the WLR. In hypertension, higher blood pressure via vasoactive peptides including endothelin 1 and angiotensin II results in peripheral arterioles vasoconstriction, smooth muscle cells apoptosis, and vascular fibrosis, resulting in remodeling^66–68^, e.g., inward eutrophic remodeling and outward hypertrophic remodeling^69^. These changes result in an increasing of the WLR. Therefore, the WLR is a sensitive marker of retinal microvascular changes indicating retinal endothelial dysfunction in systemic diseases^70^. Previous studies using AO-SLO^13^ and AO camera^39^ also showed that the blood pressure was strongly correlated with the WLR which is in keeping with our results.

This study has several limitations. First, the sample size was not large. This was partly due to the difficulty in obtaining clear images in patients with poor eye fixation or cataract in using AO camera. Second, the HbA1c was not associated with the morphological variables. Arichika et al. reported that the HbA1c was positively correlated with the retinal artery wall thickness in diabetic patients without retinopathy using AO-SLO, suggesting the AO imaging can evaluate the microvascular damage in early phase. Because we selected patients with good blood sugar control even in the PDR group may explain the absence of a significant association. Third, renal dysfunction was not considered. Chronic kidney disease is associated with endothelial dysfunction^71^, and an earlier study reported that the diameter of retinal arteries and veins decreased progressively with each stage of chronic kidney disease^72^. Fourth, the PDR group had already undergone photocoagulation. It is unclear about the morphological and blood flow changes in eyes without photocoagulation. In addition, the effect of photocoagulation on the retinal arteries is unclear. Fifth, we did not ask to patients to abstain from red meat and caffeinated beverages on the day of the examination. The consumption of red meat might have a vasodilatory effect^73^ and that of caffein may constrict ocular blood vessels^74–76^. Further longitudinal studies with multimodal imaging including renal dysfunction in a larger number of patients are needed.

In conclusion, the retinal vessel wall is thicker which leads to a narrowing of the lumen diameter and a decrease in the blood flow.
